# GPU-Based Cloud Service for Smith-Waterman Algorithm Using Frequency Distance Filtration Scheme

**DOI:** 10.1155/2013/721738

**Published:** 2013-04-03

**Authors:** Sheng-Ta Lee, Chun-Yuan Lin, Che Lun Hung

**Affiliations:** ^1^Department of Computer Science and Information Engineering, Chang Gung University, No. 259 Sanmin Road, Guishan, Taoyuan 33302, Taiwan; ^2^Department of Computer Science & Communication Engineering, Providence University, No. 200 Section 7, Taiwan Boulevard, Shalu, Taichung 43301, Taiwan

## Abstract

As the conventional means of analyzing the similarity between a query sequence and database sequences, the Smith-Waterman algorithm is feasible for a database search owing to its high sensitivity. However, this algorithm is still quite time consuming. CUDA programming can improve computations efficiently by using the computational power of massive computing hardware as graphics processing units (GPUs). This work presents a novel Smith-Waterman algorithm with a frequency-based filtration method on GPUs rather than merely accelerating the comparisons yet expending computational resources to handle such unnecessary comparisons. A user friendly interface is also designed for potential cloud server applications with GPUs. Additionally, two data sets, H1N1 protein sequences (query sequence set) and human protein database (database set), are selected, followed by a comparison of CUDA-SW and CUDA-SW with the filtration method, referred to herein as CUDA-SWf. Experimental results indicate that reducing unnecessary sequence alignments can improve the computational time by up to 41%. Importantly, by using CUDA-SWf as a cloud service, this application can be accessed from any computing environment of a device with an Internet connection without time constraints.

## 1. Introduction

The Smith-Waterman (SW) algorithm searches for a sequence database to identify the similarities between a query sequence and subject sequences [1, 2]. However, this algorithm is prohibitively high in terms of time and space complexity; the exponential growth of sequence databases also poses computational challenges [3]. Owing to the computational challenges of the Smith-Waterman algorithm, some faster heuristic solutions (e.g., FASTA [4] and BLAST [5, 6]) have been devised to reduce the time complexity yet degrading the sensitivity of alignment results.

The feasibility of using massive computational devices to enhance the performance of many bioinformatics programs has received considerable attention in recent years, especially many-core devices such as FPGAs [7–9], Cell/BEs [10–12], and GPUs [13]. The recent emergence of GPUs has led to the creation of hundreds of cores, with their computational power having exceeded one TFLOPS and NVIDIA released the CUDA programming environment [14], which allows programmers to use a common programming language (e.g., C/C++) to develop GPU-related applications to enhance the computing performance. Additionally, the feasibility of using GPUs to accelerate the SW database search problem has been widely studied, in which the pioneering work is proposed by Liu et al. [15] to develop SW algorithm using OpenGL for general-purpose GPUs (GPGPU). Following the development of the CUDA programming model, SW-CUDA [16] as the CUDA-based SW solution on GPUs could run on multiple G80 GPUs. However, SW-CUDA distributed the SW algorithm among multicore CPUs and GPUs, making it a highly dependent CPU, owing to their inability to utilize the entire computational power of GPUs. Thereafter, CUDASW++ 1.0 [17], as designed for multiple G200 GPUs, deployed all of the SW computations on GPUs to fully utilize the powerful GPUs. In contrast to previous works, CUDASW++ 2.0 [18] contributes to SW database search problem and optimizes the SIMT abstraction in order to outperform CUDASW++ 1.0. The previous research significantly improves the performance of SW algorithm; in addition, CUDASW++ 2.0 significantly reduces the search time in protein database searches.

However, when using a sequence to query a protein database, biologists do not require all results between the query sequence and all database sequences; however, the similarities are more than at a certain level. Therefore, many computations can be omitted when performing protein database searches if the minimal difference of all alignment combinations can be known in advance, allowing us to omit the extremely different combinations and retain the possible combinations in order to perform the SW alignment. Related research in recent years has heavily focused on establishing the multicore of a multicomputer system. Having received considerable attention in bioinformatics research, cloud computing integrates a large amount of computational power and storage resources, as well as provides different services through a network, such as infrastructure as a service (IaaS), platform as a service (PaaS), and software as a service (SaaS). In these cloud services, users can access desired services without location constraints. Therefore, a cloud service focuses on acquiring services via a remote connection through a network, such as the Amazon EC2 service which is an IaaS and provides various virtual machines with operating systems for users. Other service such as the Google App Engine is a PaaS cloud computing platform for developing and hosting web applications in Google-managed data centers. Other services using the SaaS platform are those such as G-mail or Dropbox services. This cloud computing platform can be viewed as an extended SaaS concept, which refers to customized software, made available via the Internet. Thus, no real computing environments in a local client do not need to be set up since these software applications do not need to ask each end user to manually download, install, configure, run, or use the software applications on their own computing environments. By using cloud services, users can even use a mobile device to complete their tasks, which could only be completed on a PC previously.

This work implements an efficient CUDA-SW program for a SW database search on GPUs. A real-time filtration method based on the frequency distance [19], referred to hereinafter as CUDA-SWf, is also designed to reduce unnecessary computations efficiently. Before the database search, a frequency vector is constructed for the query sequence and the database sequences. Frequency distances are then counted on GPUs for all combinations between query and database sequences. Frequency distance refers to the minimum difference between the query and database sequence, allowing us to record frequency distance in order to determine which combinations should be used to perform a SW alignment and then output the alignment results. Additionally, a friendly user interface (UI) is designed for the potential cloud server with GPUs. Cloud service is combined with GPU computing, in which the SaaS concept through a network is used and a UI is provided to access the service. In our test data sets, the CUDA-SWf can reduce up to 41% of the computational time by comparing with CUDA-SW. Moreover, CUDA-SWf is about 76x faster than its CPU version.

The rest of this paper is organized as follows.  Section 2 briefly describes the preliminary concepts for SW algorithm, CUDA programming model, and related works for SW algorithm on GPUs.  Section 3 then introduces the method of CUDA-SW algorithm and the implementations of the frequency filtration method. Next, Section 4 summarizes the experimental results. Conclusions are finally drawn in Section 5, along with recommendations for future research.

## 2. Related Works

### 2.1. SW Algorithm

The SW algorithm is designed to identify the optimal local alignment between two sequences by estimating the similarity score of an alignment matrix. The computation is based on a scoring matrix such as BLOSUM62 [20] or PAM250 [21] and on a gap-penalty function. Given two sequences *S*_1_ and *S*_2_ whose lengths are *l*_1_ and *l*_2_, respectively, the SW algorithm calculates the similarity score *H*(*i*, *j*) of two sequences ending at positions *i* and *j* of *S*_1_ and *S*_2_. Next, *H*(*i*, *j*) is computed, as shown in (1), for 1 ≤ *i* ≤ *l*_1_, 1 ≤ *j* ≤ *l*_2_:

(1) E(i,j)=max⁡{E(i,j−1)−Ge, H(i,j−1)−Gi−Ge},F(i,j)=max⁡{F(i−1,j)−Ge, H(i−1,j)−Gi−Ge},H(i,j)=max⁡{0,E(i,j),F(i,j), H(i−1,j−1)     +sc(S1[i],S2[j])},

where sc denotes the character substitution scoring matrix, *G*_*i*_ represents the gap opening penalty, and *G*_*e*_ refers to the gap extension penalty. A scoring matrix sc gives the substitution rates of amino acids in proteins, as derived from alignments of protein sequences.

**Figure 1:**
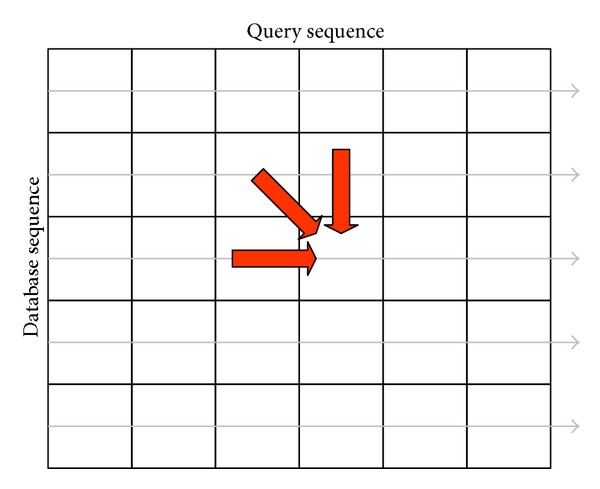
Smith-Waterman method.

The recurrences are initialized as *H*(*i*, 0) = *H*(0, *j*) = *E*(*i*, 0) = *F*(0, *j*) = 0 for 0 ≤ *i* ≤ *l*_1_ and 0 ≤ *j* ≤ *l*_2_. The maximum local alignment score refers to the maximum score in *H* function. Estimating each cell in *H* function depends on its left, upper, and upper-left neighbors, as shown by the three arrows in Figure 1. Additionally, this data dependency implies that all cells on the same minor diagonal in the alignment matrix are independent of each other and can be calculated in parallel. Thus, the alignment can be estimated in a minor-diagonal order from the top-left corner to the bottom-right corner in the alignment matrix, where calculating the minor diagonal *i* only requires the results of minor diagonals *i* − 1 and *i* − 2.

### 2.2. CUDA Programming Model (CUDA 3.2)

Compute unified device architecture (CUDA) is an extension of C/C++, in which users can write scalable multithreaded programs for GPU computing field. The CUDA program is implemented in two parts: host and device. The host is executed by CPU, and the device is executed by GPU. The function executed on the device is called a *kernel*. The kernel function can be invoked as a set of concurrently executing threads, and it is executed by *threads*. These threads are in a hierarchical organization which can be combined into *thread blocks* and *grids*. A grid is a set of independent thread blocks, and a thread block contains many threads. The grid size is the number of thread blocks per grid, and the block size is the number of threads per thread block. Threads in a thread block can communicate and synchronize with each other. Threads within a thread block can communicate through a per-block *shared memory*, whereas threads in different thread blocks fail to communicate or synchronize directly. Besides shared memory, four memory types are per-thread private *local memory*, *global memory* for data shared by all threads, *texture memory*, and *constant memory*. Of these memory types, the fastest memories are the *registers* and shared memories. The global memory, local memory, texture memory, and constant memory are located on the GPU's memory. Besides shared memory accessed by single thread block and registers only accessed by a single thread, the other memory can be used by all of the threads. The caches of texture memory and constant memory are limited to 8 KB per *streaming multiprocessor*. The optimum access strategy for constant memory is all threads reading the same memory address. The texture cache is designed for threads to read between the proximity of the address in order to achieve an improved reading efficiency. The basic processing unit in NVIDIA's GPU architecture is called the *streaming processor*. Many streaming processors perform the computation on GPU. Several streaming processors can be integrated into a streaming multiprocessor. While the program runs the kernel function, the GPU device schedules thread blocks for execution on the streaming multiprocessor. The SIMT scheme refers to threads running on the streaming multiprocessor in a small group of 32, called a *warp*. For instance, NVIDIA GeForce GTX 260, each streaming multiprocessor with 16,384 32-bit registers, has 16 KB of shared memory. The registers and shared memory used in a thread block affect the number of thread blocks assigned to the streaming multiprocessor. Streaming multiprocessor can be assigned up to 8 thread blocks. More details and other version of CUDA can be found in the CUDA programming guides. 

### 2.3. SW Algorithm on GPUs

The several platforms that the SW algorithm has been implemented on include FPGAs, Cell/Bes, and GPUs [7–18]. A query sequence compared with all database sequences is more practical than with a single sequence [22–26] (pairwise comparison). Many works have implemented the SW algorithm on GPUs. Liu et al. [13] first attempted to implement the SW algorithm on a GPU by using OpenGL. The SW algorithm has subsequently been implemented on NVIDIA graphics cards by using CUDA [14, 16]. As for database searches, many efficient methods implement the SW algorithm either by a thread called intertask parallelization or by a thread block called intratask parallelization [27]. By using intertask parallelization [27], this work calculates the similarity score of each pair of input sequences by a single thread. Additionally, a related work developed a method to perform large sequence alignment, not only a similarity score, but also alignment results, with limitations on hardware [28]. Those works improved the performance of the SW database search by using GPUs to reduce the time spent. However, increasing the efficiency of a database search is of priority concern. Performing a protein database search involves finding the most similar protein sequence in a specific database; biologists frequently perform this task. However, many low-quality results are available when performing all database comparisons, indicating the low similarity between query sequence and database sequences. The ability to identify those sequences and distinguish them from deep comparisons will significantly decrease the computational time. Additionally, the ability to qualify a filtration algorithm under this circumstance allows us to reduce computational resources and time. The most similar sequence can be obtained by filtering out the dissimilarity of characters, followed by a series of computations. When sequences are filtered, the level of filtering depends on the length of the query sequence. Longer database sequences are generally preserved to prevent containment of the query sequence. Hence, a longer query sequence implies a more efficient filtering algorithm implemented in this work.

## 3. CUDA-SW and CUDA-SWf Methods

There are two methods, CUDA-SW and CUDA-SWf, designed and implemented in this work. By integrating the frequency-based filtration method [19], CUDA-SWf performs better by reducing the comparisons than the CUDA-SW. The CUDA-SWf algorithm can be divided into three parts. 

*Part 1: Inputs Processing (Host, CPU)*. The inputs of CUDA-SWf are a query sequence and a specific protein database with a large amount of sequences. Before filtration on the device (GPU) is performed, these inputs must be processed in the following steps. 

(1) For a query sequence, CUDA-SWf records the query string and the query length, referred to hereinafter as “*Q*_*s*_” and “*Q*_*l*_,” respectively, followed by an analysis of the string character structure to construct a frequency vector (FV) for a query sequence named “*Q*_*v*_.” The *Q*_*v*_ is an integer array with 26 indices that record the frequency of each alphabet occurring in a string. Finally, *Q*_*s*_ is stored in a character array, *Q*_*l*_ is stored as an integer, and *Q*_*v*_ is stored in an integer array. 

(2) For a protein database, CUDA-SWf scans the entire database and then records the sequence string and sequence length for each database sequence, which is stored in the host memory. All database strings are stored in three one-dimensional arrays, referred to hereinafter as “*D*_*s*_,” “*D*_*l*_,” and “*D*_*e*_,” respectively. Notably, *D*_*s*_ stores all characters of each database sequence; *D*_*l*_ stores the length of each database sequence in *D*_*s*_; *D*_*e*_ stores the start position of each database sequence in *D*_*s*_. The sequence length must be shorter than 2,000 characters; owing to that when executing the SW algorithm, some data must be stored in the local memory; in addition, local memory size for each thread is limited. In this step, CUDA-SWf does not construct the frequency vector for each database sequence; owing to that the database contains a large amount of sequences and the cost is high for constructing the frequency vector for each database sequence on the host (sequentially). CUDA-SWf constructs a frequency vector for each database sequence on the device (GPU) when executing the filtration method (run time filtration method). 

*Part 2: Implementation of the Frequency Filtration Method (Device, GPU)*. Inputs on the host should first be transferred from the host to the device. Because the query data are used and not updated, the query string, *Q*_*s*_, query length, *Q*_*l*_, and query frequency vector, *Q*_*v*_, are stored in the constant memory. The size of database sequence data (*D*_*s*_, *D*_*l*_, and *D*_*e*_) is too large and stored in the global memory. 

When implementing the filtration method, assume that two similar sequences found by SW algorithm may have a certain number of the same characters. As restated, counting the different characters can help to filter out the dissimilar sequences by the enormous difference among character structures. Counting the different characters for each database sequence and query sequence is relatively easy; CUDA-SWf allows a thread to analyze the difference between the query and a database sequence. To analyze the differences between query and database sequences, each thread must construct an FV for a database sequence named “*D*_*v*_.” Similar to *Q*_*v*_, the *D*_*v*_ value of each database sequence is also an array with 26 indices to store the appeared frequency of each alphabet. Next, counting the sum of the differences between the number of each alphabet in the *D*_*v*_ and *Q*_*v*_ allows us to calculate the differences in their character structure, which is called *frequency distance* (FD). Frequency distance refers to the minimum differences between two sequences. The details of FV and FD can be found in the literature [19]. 

Finally, a variable “mismatch percentage (MP)” is available to determine whether to perform SW comparisons. Notably, MP refers to the allowed maximum differences ratio between a query and a database sequence; a small value implies a strict filter due to the small FD allowed; otherwise, it implies loose with large FD. When the FD value between a query sequence and a database sequence is greater than MP, it refers to a situation in which the maximum similarity ratio of these two sequences is not satisfied, and this database sequence can be filtered out. When the FD value between a query sequence and a database sequence is lower than MP, it refers to a situation in which the maximum similarity ratio of these two sequences may be satisfied, and this database sequence should make a SW comparison with the query sequence. An attempt is made to prevent database sequences from having too long length, which would make the sequences filtered out due to the large value of FD. When calculating FD, if *D*_*l*_ is longer than *Q*_*l*_, CUDA-SWf will consider that this database sequence must be compared with the query sequence by a SW algorithm. In doing so, a situation can be avoided in which the query sequence is a local (partial) sequence of the database sequences. 

**Figure 2:**
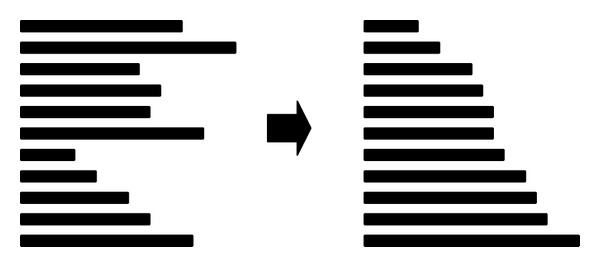
Sorting of the selected sequence to assemble the sequences of a similar length for an improved load balance.

**Figure 3:**
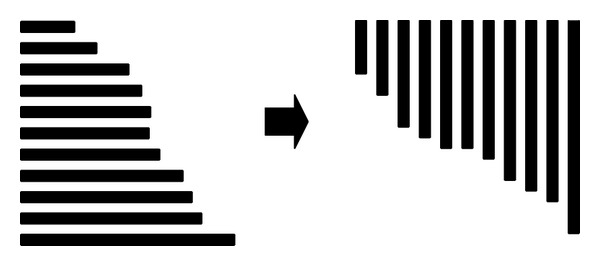
Memory patterns of sequences in the global memory.

*Part 3: SW Comparison (Device and Host)*. Following selection of the frequency filtration method, CUDA-SWf performs the SW comparison for each selected database sequence with the query sequence. CUDA-SWf uses a thread to make a SW comparison that is called intertask parallelization. To improve the load balance and memory access pattern, CUDA-SWf moves the selected database sequences to the host memory before making SW comparisons for sorting and rearranging the memory pattern for selected database sequences for two subjects: (i) improved load balance for each thread in the same thread block and (ii) coalesced global memory access [17]. In the CUDA programming model, a thread block occupies the resource of a streaming multiprocessor (SM) until all threads in the same thread block complete their computations. To improve the load balance for interftask parallelism, CUDA-SWf must ensure that all threads in the same thread block are assigned a similar length of sequences to achieve a better load balance by sorting the database sequences to assemble the sequences of a similar length, as shown in Figure 2. In order to simply the work in CUDA-SWf, the sorting is performed on CPU. After sorting the database sequences, CUDA-SWf converts the memory configuration from the row major to the column major, as shown in Figure 3 in order to coalesced global memory access. Therefore, all threads in a thread block can access sequences in a continuous memory space. During implementation of the SW algorithm, the alignment sequences must be stored in the global memory and then moved to the local memory of a multiprocessor. The Fermi architecture has per-SM L1 cache and unified L2 cache to service the load/store to global memory; to maximize the performance of cache memory, all threads in the same warp should access the alignment data in global memory to maximize the efficiency of cache memory.

**Figure 4:**
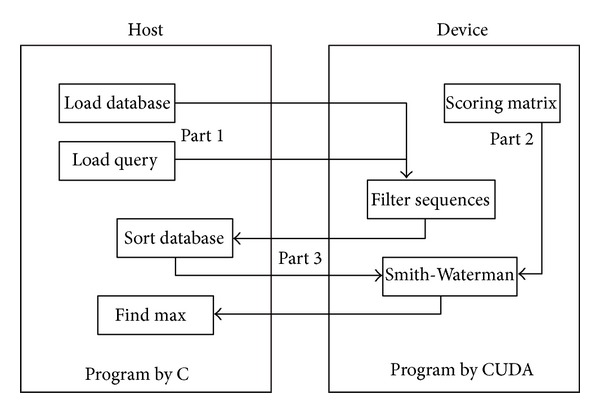
Flowchart of CUDA-SWf.

To output the alignment result by the trace back path, the original SW comparison must calculate and store the values in a *M* × *N* matrix (*M* denotes the query length and *N* represents the selected database sequence length), explaining why its space complexity is O(*N* ^2^), assuming that *M* is equal to *N*. In this work, CUDA-SWf only reports the similarity score, not alignment result, and does not need to record the trace back path, explaining why its runtime space complexity to each thread can decrease O(2*N*) and suitable for using the intertask parallelization. Because each thread service requires a selected sequence comparison to perform the query sequence, the shared memory cannot load all alignment data. CUDA-SWf thus stores the alignment data of each thread in the local memory. In the Fermi architecture, it is still efficient to store data in the local memory due to L1/L2 cache. Notably, performance of the local memory is not far away from that of the shared memory and is even better than that of the shared memory when the bank conflict occurs in shared memory. The SW comparison of each thread can be divided to three steps: (i) create alignment data: when the comparison is initiated, each thread must create two integer arrays *A* and *B*, in which size denotes the length of a selected sequence and stored in the local memory. Owing to that the size limitation of local memory is 16 KB per-thread and the maximum length of database sequence is 2,000. (ii) Row by row comparison: CUDA-SWf can only output the alignment similarity score. Array *A* is first assigned the value of 0 and, then, each row cell can be calculated simultaneously and the calculated score is stored in array *B*. Next, the values in array *B* are moved to array *A*. Finally, the next row is calculated until all comparisons are finished. (iii) Store the maximum score and final output: when each row comparison is completed, CUDA-SWf confirms the maximum score and records it; finally, CUDA-SWf stores the maximum score in the global memory and, then, moves it to the host memory and finally outputs the database sequences that are similar to the query sequence. The flowchart of CUDA-SWf is shown in Figure 4. The CUDA-SW method is similar to CUDA-SWf without the frequency filtration method.

## 4. Results

CUDA-SWf was implemented on NVIDIA Tesla C2050 (G400 GPU) with 14 streaming multiprocessors, consisting of 448 CUDA cores and 2.5 GB RAM. The host (CPU) is Intel Xeon E5506 2.13 GHz with 12 GB RAM running on Linux operation system. The protein sequence database was human protein database downloaded from NCBI (http://www.ncbi.nlm.nih.gov/); the query sequences were selected from the H1N1 virus database from the Influenza Virus Resource from NCBI (http://www.ncbi.nlm.nih.gov/genomes/FLU/FLU.html). The testing data sets include the following: (1) 32,799 protein sequences of human with an average length of 555 as the database, and (2) H1N1 virus protein sequences that were randomly selected from the NCBI H1N1 virus database, and the length brackets are 100, 200, 300, 400, 500, 600, and 700 as query sequences. After deleting the protein sequence with length larger than 2,000, there are 32,133 human sequences used in the following tests. The gap open penalty was set to 10.0; the gap extension penalty was set to 2.0; the scoring matrix was BLOSUM62. Next, the MP was set to 10%, 30%, 50%, and 100%, implying the number of different characters between query sequence and database sequences. When the MP is set to 100%, it means that no filtration method is used in CUDA-SWf. The number of threads in a thread block is set to 128; the number of thread blocks depends on the number of sequences that must be compared with query sequences.

Table 1 shows the overall computation time of CPU version of SW algorithm, CUDA-SW, and CUDA-SWf for human protein database and H1N1 virus sequences under various query sequence lengths with MP of 10%. The overall computation time of CUDA-SWf is the sum of computation time in each part. Table 1 indicates that the proposed frequency filtration method can reduce up to 46% of the computation time by filtering out the database sequences in which the minimum different ratio exceeds 10%. Besides, there are two observations in Table 1. First, the computation time increases when the query sequence (H1N1 virus) length increases. The time complexity of SW algorithm is proportional to the query sequence length. Second, the improved ratio increases when the query sequence length increases. The reason is that the number of filtered database sequences is few when the query sequence length is short. When the query sequence length is short, most of database sequences have larger length than it, and they should make SW comparisons in Part 3 of CUDA-SWf.

Table 2 shows the overall computation time of CUDA-SW for human protein database and H1N1 virus sequences under various MPs with the query length of 700. Table 2 indicates that the number of selected database sequences decreases when the MP decreases. When MP is 100%, there are 32,133 human protein sequences selected to make following SW comparisons; when MP is 10%, only 21.8% of 32,133 human protein sequences can be selected. Therefore, the computation time of CUDA-SWf is reduced from 8.27 to 4.4 (near to 47% improved ratio). When doing the filtration method, extra computation time is needed for CUDA-SWf to construct FV and calculate FD for each database sequence and sorting database sequences on the host. From Table 2, the best score can be found by CUDA-SWf under various MPs. It implies that the frequency filtration method in CUDA-SW is suitable for database search problem. Besides, in Table 2, the worst score found by CUDA-SWf when MP is 10% is closer to that when MP is 100%. This phenomenon indicates that a selected database sequence with low FD may have large difference to a query sequence. Therefore, the FD can be used to filter out the dissimilar sequences; however, it cannot be used to determine the similarity score. 

**Figure 5:**
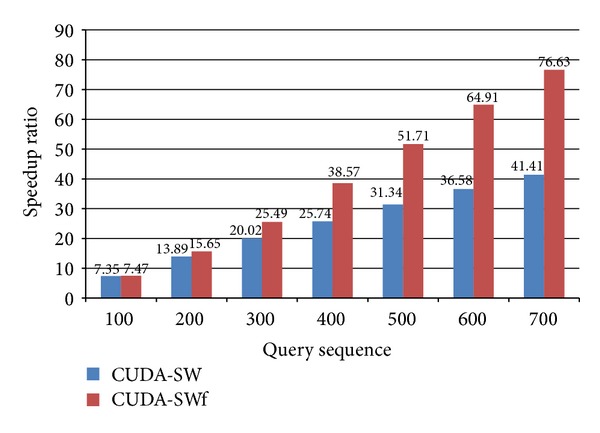
Speedup ratio of CUDA-SW, and CUDA-SWf with MP (10%).

Figure 5 shows the speedup ratio of CUDA-SW and CUDA-SWf by comparing with CPU version of SW algorithm for Human protein database and H1N1 virus sequences under various query sequence lengths with MP of 10%. From Figure 5, the speedup ratios of CUDA-SW range from 7x to 41x; the speedup ratios of CUDA-SWf range from 7x to 76x. The improvement is significant when the query sequence length is larger than 400 due to large number of database sequence filtered out.

**Figure 6:**
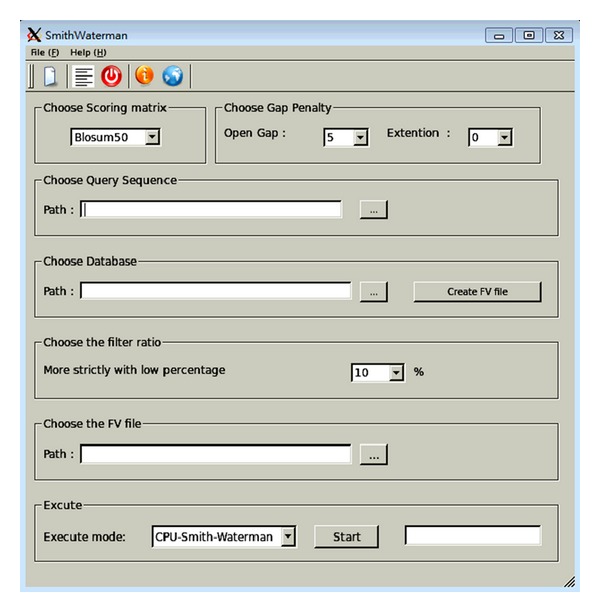
Workbench of CUDA-SWf.

For the user interface, this work constructs a workbench for CUDA-SWf with QT Creator 2.4.1 (http://qt.nokia.com/products) on Ubuntu 10.04.1, as shown in Figure 6. As a cross-platform application framework, QT is used to design the same UI for different operating systems then through a network, which transfer the input data to a cloud server. Figure 6 reveals 7 steps to run the CUDA-SWf method.

**Step 1 (select the scoring matrix)**

Notably, the scoring matrix is needed when doing the SW comparison. Five matrices are provided in this work: Blosum50, Blosum62, Blosum80, PAM100, and PAM250.

**Step 2 (select the gap penalty)**

Users can select the desired penalty. The open gap penalty range is 5~20, and the gap extension penalty range is 0~10.

**Step 3 (select query sequence)**

Users select a sequence as a query sequence. If a new query file is available, a new file can be created using File(F)->New(N).

**Step 4 (select the database)**

A database can be selected or created by the button “Create FV file.” Users can download the database from NCBI. Also, a new database can be created using the button, in order to implement the frequency filtration method. This button creates two files: the first one is the new database sorted by length; and the second one is the FV file by the new database file.

**Step 5 (select the filter ratio (MP))**

Filter ratio can allow users to determine how strict the CUDA-Swf is used with the filtration method. Users can choose from 10%~100%. 10% refers to the sequences with those with more than 90% similarity to be computed.

**Step 6 (select the FV file)**

Users select the FV file to be created at Step 4, which helps to execute the frequency filtration method.

**Step 7 (execute CUDA-SWf)**

**Figure 7:**
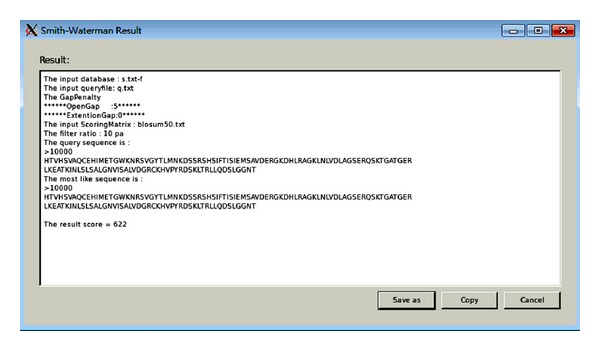
Result window of CUDA-SWf.

Two modes can be selected, CPU or GPU. GPU version requires CUDA. Following their execution, the result window is shown (Figure 7). The empty text line displays a message with some errors.

The workbench for CUDA-SWf is freely available to download at http://163.25.101.18/~ppcb/main/research/CUDASWF.html.

## 5. Conclusions

This work designs and implements a novel CUDA-SWf method to solve the Smith-Waterman database search problem with a frequency-based filtration method and CUDA. The proposed method focuses on the intratask parallelization to calculate the frequency distance and perform Smith-Waterman comparisons on a single GPU. Experimental results demonstrate that the proposed CUDA-SWf method achieves up to 76x speedup ratio under a single GPU for the computation time. Moreover, CUDA-SWf can improve the computational time by up to 41% than CUDA-SW without the frequency filtration method. These results demonstrate that CUDA-SWf can accelerate the Smith-Waterman algorithm on GPUs, and the novel idea is still worth to be designed and proposed in order to enhance the performance of CUDA applications.

## Figures and Tables

**Table 1:** Overall computation time of CPU version of SW algorithm, CUDA-SW, and CUDA-SWf with MP (10%).

H1N1 virus query sequence length (bp)	CPU version of SW (second)	CUDA-SW (second)	CUDA-SWf (second)	Improved ratio (CUDA-SWf versus CUDA-SW)
100	49.91	6.79	6.68	1.62%
200	97.84	7.04	6.25	11.22%
300	145.6	7.27	5.71	21.46%
400	193.62	7.52	5.02	33.24%
500	243.56	7.77	4.71	39.38%
600	293.43	8.02	4.52	43.64%
700	343.31	8.29	4.48	45.96%

**Table 2:** Overall computation time of CUDA-SWf with query sequence length (700).

MP	Number of selected database sequences	Differences (worst score)	Differences (best score)	CUDA-SWf (second)
100%	32,133	3,542	1,169	8.27
50%	17,913	3,542	1,169	5.77
30%	8,578	3,536	1,169	4.63
10%	7,007	3,525	1,169	4.4
